# Enzyme-Activated Self-Assembling Peptides Mimicking Adiponectin Multimers for Nonalcoholic Fatty Liver Disease Therapy

**DOI:** 10.1021/acscentsci.5c02405

**Published:** 2026-02-11

**Authors:** Zenghui Li, Shuangdi Duan, Zihao Zhu, Hong Han, Nong Qin, Qiaoqiao Ji, Dan Yuan, Junfeng Shi

**Affiliations:** † Hunan Provincial Key Laboratory of Animal Models and Molecular Medicine, State Key Laboratory of Chemo/Bio-Sensing and Chemometrics, School of Biomedical Sciences, 12569 Hunan University, Changsha, Hunan 410082, China; ‡ 74626 The First Affiliated Hospital of Guangxi Medical University, Nanning, Guangxi 530021, China; § Shenzhen Research Institute, 12569 Hunan University, Shenzhen, Guangdong 518000, China

## Abstract

Adiponectin is a multifunctional adipokine that regulates metabolic homeostasis, particularly lipid metabolism, through activation of adiponectin receptors (AdipoRs). Its high molecular weight (HMW) form exhibits the greatest biological activity, yet therapeutic peptides derived from adiponectin typically exist as monomers or aggregates, limiting their efficacy. To mimic the multimeric architecture of adiponectin and enhance peptide efficacy, we developed two alkaline phosphatase (ALP)-activated self-assembling peptides, 1P and 2P, based on a conserved adiponectin sequence (^148^GKFH­CNIPGL­YYFAY^162^). These peptides undergo in situ self-assembly into stable nanofibers in ALP-overexpressing liver tissue, enhancing structural stability and receptor engagement. The assembled peptides effectively bind AdipoRs and reduce lipid accumulation in vitro. In a high-fat diet (HFD)-induced nonalcoholic fatty liver disease (NAFLD) mouse model, treatment with these peptides led to significant reductions in body weight, blood glucose levels, and hepatic steatosis. Transcriptomic analysis further revealed modulation of key pathways involved in inflammation, lipid synthesis, and metabolism. This study offers a promising strategy for mimicking multimeric adipokine structures and advancing peptide-based therapeutics for NAFLD.

## Graphical abstract

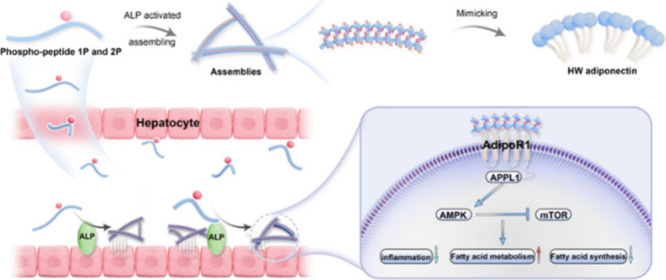

## Introduction

Nonalcoholic fatty liver disease (NAFLD) is a prevalent chronic liver disorder characterized by excessive hepatic fat accumulation independent of significant alcohol consumption.^1^ NAFLD encompasses a broad spectrum of pathological conditions, ranging from simple steatosis to nonalcoholic steatohepatitis (NASH), which involves hepatocellular injury, inflammation, and varying degrees of fibrosis.^2^ It is closely associated with metabolic syndrome, obesity, insulin resistance, and type 2 diabetes, and poses a significant risk for progression to cirrhosis and hepatocellular carcinoma.^3,4^ Owing to its increasing prevalence and limited treatment options, NAFLD has emerged as a major public health concern, necessitating the development of novel therapeutic strategies targeting hepatic metabolism and inflammation. Current therapeutic efforts for NAFLD focus on targeting key metabolic and inflammatory pathways, including peroxisome proliferator-activated receptor (PPAR) agonists,^5^ farnesoid X receptor (FXR) agonists,^6^ ALKBH5 Inhibitor,^7,8^ and transforming growth factor-β.^9^ However, most candidate drugs have shown limited efficacy and remain in clinical development stages, underscoring the need for more effective therapeutic modalities.^10^

Adiponectin, a major adipokine secreted by adipose tissue, plays a crucial role in regulating glucose and lipid metabolism, exerting anti-inflammatory, antifibrotic, and insulin-sensitizing effects primarily through interaction with its receptors AdipoR1, followed by activation of the AMPK and PPAR-α signaling pathways.^11–13^ Clinical and preclinical studies have shown that low circulating adiponectin levels are strongly associated with the onset and severity of NAFLD and NASH.^14,15^ Conversely, restoring adiponectin levels via dietary or pharmacological interventions can mitigate disease progression.^16^ These findings suggest that adiponectin serves as a promising drug candidate for NAFLD and NASH treatment. Notably, adiponectin exists in multiple oligomeric forms—low-, medium-, and high-molecular weight (LMW, MMW, and HMW)—with the HMW form exhibiting the highest biological activity and showing a stronger association with NAFLD and NASH.^17^ Recently, a highly conserved 15-residue segment of adiponectin (^148^GKFH­CNIPGL­YYFAY^162^) has been identified as a bioactive core that mimics the function of full-length adiponectin.^18–22^ Despite several peptide-based therapeutics derived from this motif, their clinical utility has been hampered by low stability and limited efficacy—likely due to their monomeric nature and inability to mimic the multimeric structure of functional adiponectin.^23–25^

Enzyme-responsive self-assembly has been widely applied in cancer therapy and tissue engineering;^26,27^ however, its use to mimic protein multimer and recapitulate their biological functions remains largely unexplored. Adiponectin, which naturally forms higher-order oligomers, provides an ideal model for functional simulation via peptide-based, enzyme-activated self-assembly. These engineered assemblies not only reproduce the multimeric architecture critical for adiponectin activity but also enhance molecular stability and improve druggability,^28^ representing a significant advancement over prior peptide mimetics. Thus, we leveraged molecular self-assembly—an intrinsic biological process mediated by noncovalent interactions—to develop enzyme-activated, self-assembling peptides that structurally and functionally mimic HMW adiponectin.^29–34^ Specifically, we reported two phosphorylated peptides (**GLYYpF** and **NvaLYYpF**, denote **1P** and **2P**) that undergo alkaline phosphatase (ALP)-triggered dephosphorylation and subsequent nanofiber formation in ALP-rich liver tissue (Figure 1). These peptides showed robust AdipoR1 binding following activation, improved stability against enzymatic degradation, and potent lipid-lowering activity in vitro and in vivo. In HFD-induced NAFLD mouse models, both peptides alleviated hepatic steatosis, normalized blood glucose, and modulated lipid metabolism through AMPK activation and mTOR inhibition. Transcriptomic profiling further revealed suppression of lipogenesis and inflammatory pathways, providing molecular insights into their therapeutic mechanism. Taken together, this work introduces a modular and activated nanotherapeutic platform that not only addresses the limitations of adiponectin-mimetic peptides but also offers a versatile strategy for targeting metabolic disorders through supramolecular peptide design.

**Figure 1:**
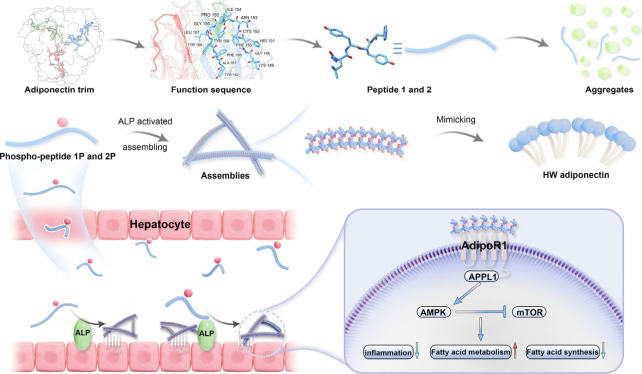
Schematic representation of alkaline phosphatase (ALP)-triggered self-assembly of adiponectin-mimetic peptides targeting AdipoR1, leading to reduced hepatic lipid accumulation and inflammation in NAFLD.

## Results and Discussion

### Self-Assembled Peptide Design and Characterization

According to previous studies, we found that the five core amino acids (GLYYF) within the 15-mer adiponectin sequence share high similarity with the typical self-assembling motif GFFY, suggesting similar assembly and physicochemical properties.^35^ Notably, **GLYYF** retains the biological activity of adiponectin.^21,22^ In addition, substituting glycine (**Gly**) with the non-natural amino acid norvaline (**Nva**) has been shown to enhance both bioactivity and affinity. The increased hydrophobicity of **Nva** promotes stronger self-assembly behavior, while its non-natural structure offers greater resistance to enzymatic degradation, thus improving the stability and function of the peptide.^36^ Thus, we synthesized two peptides, **GLYYF** (denotes **1**) and a non-natural mutation variant **NvaLYYF** (denotes **2**), along with their phosphorylated analogs, **1P** and **2P** to enable enzyme-activated self-assembly in ALP-rich liver tissue under pathological conditions (Figures S1–S3).^37,38^ Moreover, phosphorylation on tyrosine (Y) at the fourth amino acid was introduced to suppress AdipoR1 binding by altering the side chain polarity of **Y**, rendering both **1P** and **2P** biologically inactive prior to ALP catalysis.^22^

We then examined their ALP-activatable properties and characterized their self-assembly behavior. The dephosphorylation kinetics revealed that both **1P** and **2P** underwent complete dephosphorylation within 8 h of ALP incubation (Figure 2a,b), indicating their responsiveness in ALP-overexpressing cell lines. Transmission electron microscopy (TEM) revealed that **1P** assembled into nanofibers, while **2P** transitioned from nonordered nanostructures to well-defined filamentous bundles upon ALP treatment (Figure 2c,d). The peptide solutions remained clear in phosphate buffer (PBS, pH = 7.4), likely due to the hydrophilicity of the phosphate groups, which also improved solubility prior to ALP addition. To investigate the effect of phosphate groups on assembly, we measured the critical aggregation concentration (CAC) of all peptides. The results showed that **1P** had a CAC of 932.2 μM, which decreased to 125.9 μM for **1**. Similarly, the CAC of **2P** decreased from 955.0 μM to 72.6 μM for **2** (Figure 2e). These results suggest that phosphorylation enhances peptide solubility and that ALP-triggered dephosphorylation promotes more efficient self-assembly. To evaluate the multivalency of peptide assemblies, we used gel permeation chromatography (GPC) to determine the molecular weight distribution.^39,40^ Following ALP treatment, **1P+ALP** and **2P+ALP** exhibited broad molecular weight distributions (∼400–30 000 Da and ∼20 000–100 000 Da, respectively), whereas their nonphosphorylated counterparts remained in the lower range (**1**: ∼300–4000 Da; **2**: ∼1,500–4000 Da), Similarly, phosphorylated peptides incubated without ALP also remained within low–molecular-weight ranges (**1P**: ∼700–2000 Da; **2P**: ∼700–3000 Da) (Figure S4). This increase in molecular size upon ALP treatment suggests the formation of multimeric assemblies that potentially mimic the structural and functional properties of adiponectin. Because self-assembly can enhance proteolytic resistance, we compared the stability of monomeric and assembled peptides in the presence of proteinase K. The assembled peptides (**1P** and **2P**) remained stable for over ∼150 min, while nonphosphorylated peptides degraded within ∼60 min in vitro degradation assays (Figure 2f). To assess the biocompatibility of peptides, hemolysis and cell viability assays were conducted. None of the peptides induced significant hemolysis in red blood cells (Figure S5a), demonstrating good biocompatibility. These results confirm that phosphorylated peptides can undergo ALP-triggered self-assembly into stable nanofibers, thereby enhancing both bioactivity and proteolytic resistance for potential therapeutic applications.

**Figure 2:**
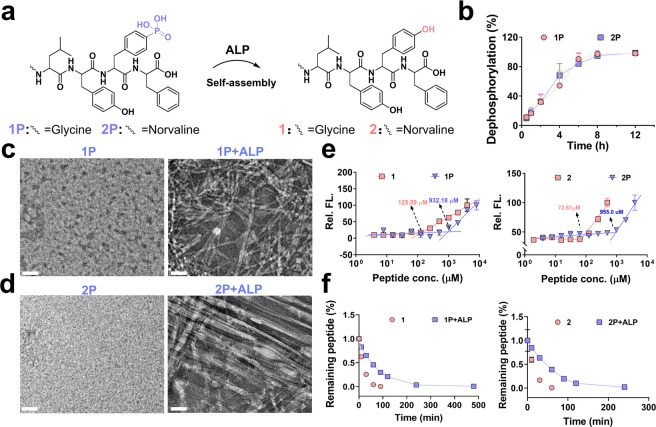
Physical characterization of phosphorylated peptides derived from adiponectin. (a, b) Dephosphorylation kinetics of 300 μM **1P** (a) and **2P** (b) in the presence of 5 U/mL ALP in PBS buffer. (c, d) TEM images of **1P** (c) and **2P** (d) after ALP treatment; scale bar: 50 nm. (e) CAC measurement of **1P** vs **1** and **2P** vs **2** in PBS. (f) Proteolytic stability of 300 μM **1P**, **1**, **2P**, and **2** over time in the presence of proteinase K.

### Enzyme-Activated Peptide Self-Assembly and AdipoR1 Binding

To investigate the self-assembly behavior of peptide **1P** and **2P** in cellular environment and their interaction with adiponectin receptors 1 (AdipoR1), NBD-labeled peptides were incubated with the Huh7 cells, which often used as a model for studying liver function. Prior to cell imaging, MTT assays were performed to determine a nontoxic concentration for cellular experiments. The results indicated a slight cytotoxicity for both **1P** and **2P** at 500 μM toward huh7 and HepG2 cells (Figure S5b). ALP activity assays revealed that Huh7 hepatoma cells exhibit significantly higher ALP activity compared to Normal Human Dermal Fibroblasts (NHDF) (Figure S6). Confocal fluorescence imaging revealed pronounced green fluorescence from NBD-labeled peptides, which was significantly diminished upon ALP inhibition (Figure 3a), indicating ALP-dependent peptide activation and self-assembly. This observation was further corroborated by flow cytometry, which showed a significant reduction in peptide-associated fluorescence following 1 mM levamisole treatment (Figure S7a).^41^ To examine the interaction between the peptides and AdipoR1, FITC-labeled peptides were coincubated with cells and analyzed for colocalization with AdipoR1. As shown in Figure 3c, partial overlap between green (FITC-peptides) and red (AdipoR1) fluorescence suggests direct interaction between the assembled peptides and AdipoR1. Co-localization analysis of peptides **1** and **2** with AdipoR1 was conducted by masking overlapping pixels from the green (peptide) and red (AdipoR1) channels, revealing partial spatial overlap between the peptides and AdipoR1 (Figure S8). Furthermore, Huh7 cells were transfected with shRNA targeting AdipoR1. Western blotting confirmed effective knockdown of AdipoR1 (Figure S9). Following AdipoR1 silencing, confocal imaging and flow cytometry analysis showed significantly reduced cellular binding of FITC-labeled peptides, whereas cells treated with control plasmids retained strong fluorescence signals (Figure 3b and Figure S7b). These findings indicate that the designed peptide bind to cells in an AdipoR1-dependent manner.

**Figure 3:**
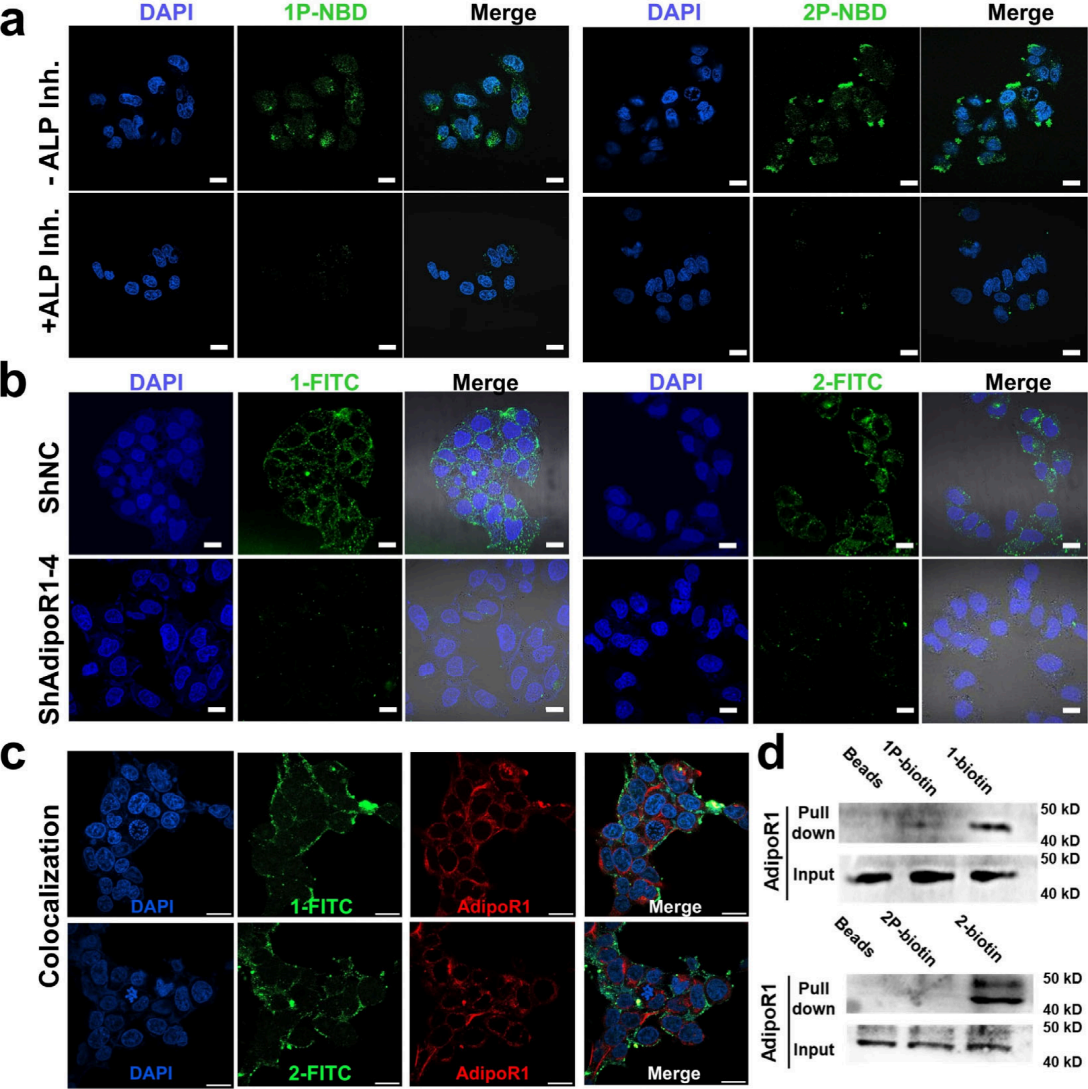
Enzyme-activated peptide self-assembly and AdipoR1 binding. (a) Confocal images of huh7 cells after incubation with 100 μM NBD-labeled **1P** and **2P** for 3 h in the presence and absence of ALP inhibitor levamisole (1 mM), showing ALP-dependent self-assembly; scale bar 20 μm. (b) Confocal images of 50 μM FITC-labeled **1** (left) and **2** (right) incubated with Huh7 cells transfected with either control shNC or AdipoR1-targeting shRNA (shAdipoR1), demonstrating peptide binding to AdipoR1 on the cell surface; scale bar 20 μm. (c) Immunostaining for AdipoR1 to detect colocalization with FITC-labeled **1** and **2**; scale bar 20 μm. (d) Pull-down experiments investigating the interaction between biotin-labeled peptides and AdipoR1.

To further validate this interaction, pull-down assays were performed using biotin-labeled peptides with Huh7 cell lysates. Notably, only the nonphosphorylated peptides (**1** and **2**) successfully pulled down AdipoR1 (Figure 3d). In contrast, the phosphorylated variants (**1P** and **2P**) showed no detectable binding, confirming that phosphorylation at the tyrosine residue blocks AdipoR1 recognition. The diminished binding of **1P** and **2P** can be restored via ALP-mediated dephosphorylation. Notably, a phosphatase inhibitor cocktail was added to the cell lysate during the pull-down assay to prevent the dephosphorylation of **1P** and **2P**, thereby avoiding potential false-positive results. In addition, microscale thermophoresis (MST) analysis was performed to quantitatively assess the binding of the peptides to AdipoR1. Peptides **1** and **2** exhibited moderate binding affinities, with *K*_d_ values of 45.28 μM and 117.29 μM, respectively. In contrast, the phosphorylated peptides **1P** and **2P** showed no detectable binding under the same conditions, indicating that phosphorylation effectively abolishes their interaction with AdipoR1 (Figure S10). Collectively, these results establish that ALP-triggered dephosphorylation is a prerequisite for peptide self-assembly and AdipoR1 binding, thereby providing mechanistic validation of these supramolecular peptides as an enzyme-activated adiponectin mimetic.

### Assembling Peptide Alleviates Lipid Accumulation and Oxidative Stress in Hepatocyte

To evaluate the therapeutic potential of the adipo-peptides in reducing hepatic lipid accumulation, an oleic acid (OA)-induced lipid overload model was established in Huh7 cells. Lipid accumulation was successfully induced using 800 μM OA, as evidenced by the upregulation of adipose differentiation-related protein (ADRP) (Figure 4a). Following OA induction, cells were treated with these peptides, and intracellular triglyceride (TG) and total cholesterol (TC) levels were quantified. Statistical analysis revealed that the adipo-peptide significantly reduced both TG and TC levels after 48 h of treatment, as shown in Figure 4b. Notably, both the phosphorylated peptides (**1P**, **2P**) and their nonphosphorylated counterparts exhibited greater lipid-lowering effects compared to the negative control peptide, **GLAAF**. BODIPY staining, analyzed via confocal laser scanning microscopy (CLSM) and flow cytometry, further confirmed the lipid-reducing effect. Flow cytometry revealed that lipid droplet content in the adipo-peptide-treated group nearly returned to baseline levels, compared to the cells treated with OA alone (OA group) (Figure 4c). CLSM images showed a marked reduction in both the size and number of lipid droplets (Figure 4d). Given that NAFLD involves lipid accumulation, oxidative stress, and inflammation, we next examined the impact of peptide treatment on redox balance. Reactive oxygen species (ROS) staining demonstrated a notable decrease in green fluorescence intensity in the peptide-treated cells, suggesting reduced oxidative stress—likely a result of decreased lipid burden (Figure 4e). This effect was further validated by flow cytometry quantification, which demonstrated a significant reduction in ROS levels in peptide-treated cells compared with both the control peptide and PBS-treated groups (Figure S11). Western blot analysis revealed that peptide treatment elicited a concentration-dependent activation of the AMPK signaling pathway, accompanied by a marked reduction in ADRP expression. The effects become particularly pronounced at 200 μM peptide concentration (Figure 4f). Since AMPK activation is a well-established mechanism for suppressing lipogenic pathways and reducing lipid droplet accumulation in metabolic tissues, these findings indicate that the designed adipo-peptides not only enhance AMPK activity but also attenuate lipid storage.^42^ Together, these results support the ability of these peptides to mitigate lipid accumulation and oxidative stress in hepatocyte models, highlighting their potential as therapeutic candidates for NAFLD and related metabolic liver diseases.

**Figure 4:**
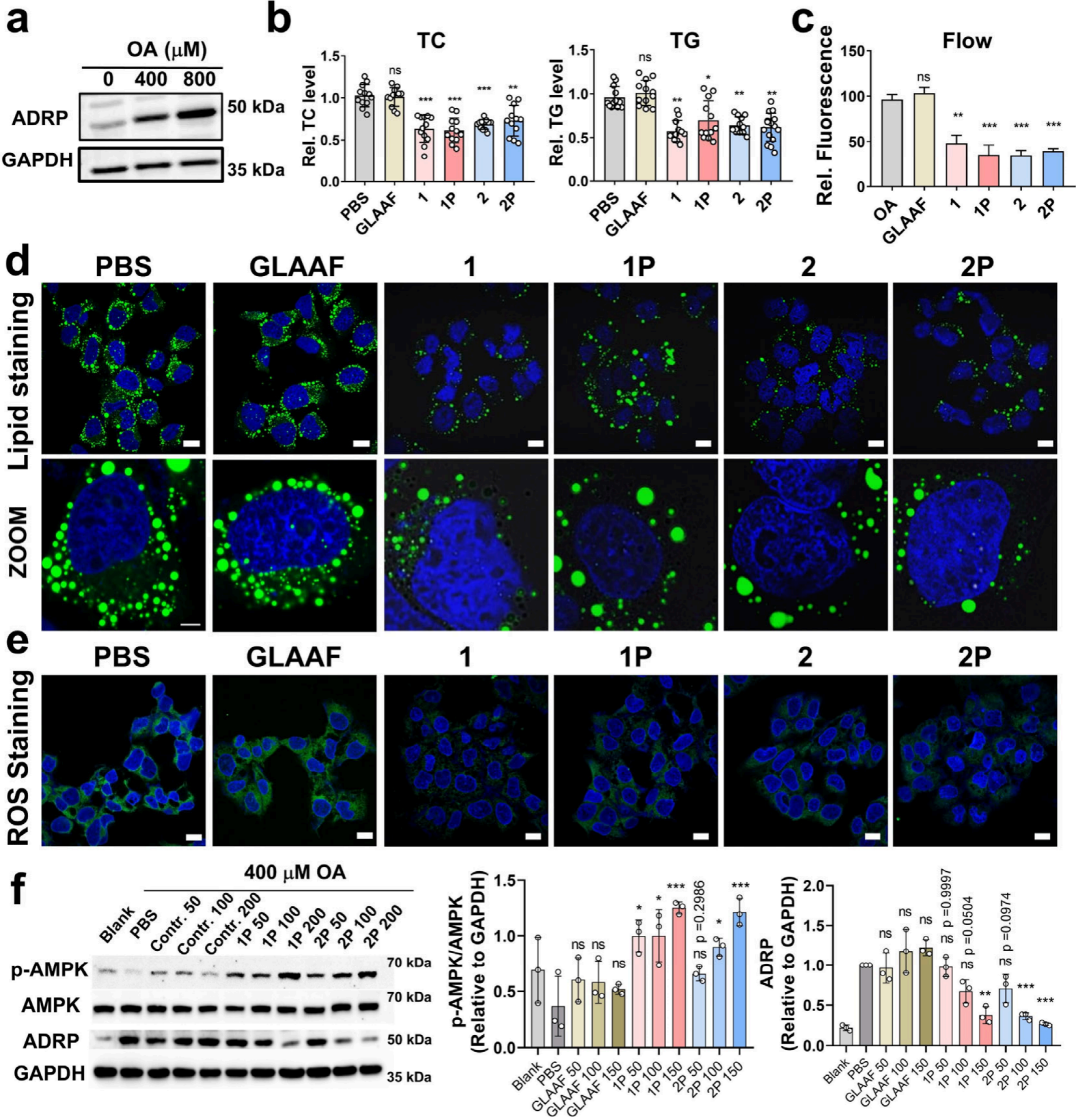
Self-assembling peptides alleviate lipid accumulation and oxidative stress in hepatocytes. (a) WB analysis of ADRP expression in Huh7 cells treated with 400 μM and 800 μM OA to induce lipid accumulation. (b) TC and TG quantification of OA-treated Huh7 cell after incubation with peptides (*n* = 12). (c) Flow cytometer (*n* = 3) and (d) confocal microscopy analysis of lipid droplets in OA-treated Huh7 cells following peptide treatment; scale bar 10 μm; zoom (5 μm). (e) ROS levels detected by fluorescence staining following peptide treatment; scale bar 10 μm. (f) Western blot and quantification analysis showing that peptides reduce lipid accumulation by activating AMPK (*n* = 3). Mean ± SD; *p < 0.05; **p < 0.01; ***p < 0.001; ns, no significance.

### Enzyme-Activated Self-Assembling Peptides Relieve Lipid Accumulation via AdipoR1

To further elucidate the role of ALP in peptide-mediated lipid reduction, we assessed whether inhibiting ALP would compromise the lipid-lowering activity of **1P** and **2P**. In OA-induced Huh7 cells, BODIPY staining revealed strong green fluorescence indicating lipid accumulation. Treatment with **1P** and **2P** markedly reduced this signal, consistent with lipid clearance. Consistent with the confocal microscopy results, co-treatment with an ALP inhibitor restored lipid droplet-associated fluorescence, indicating that ALP inhibition blocked peptide activation by preventing dephosphorylation (Figure 5a). Quantitative flow cytometry analysis further corroborated this trend (Figure S12). Quantification assays of TG and TC, along with Western blot analysis of ADRP expression, further confirmed that ALP activity is indispensable for the lipid-reducing effect of these peptides (Figure 5b,c).

**Figure 5:**
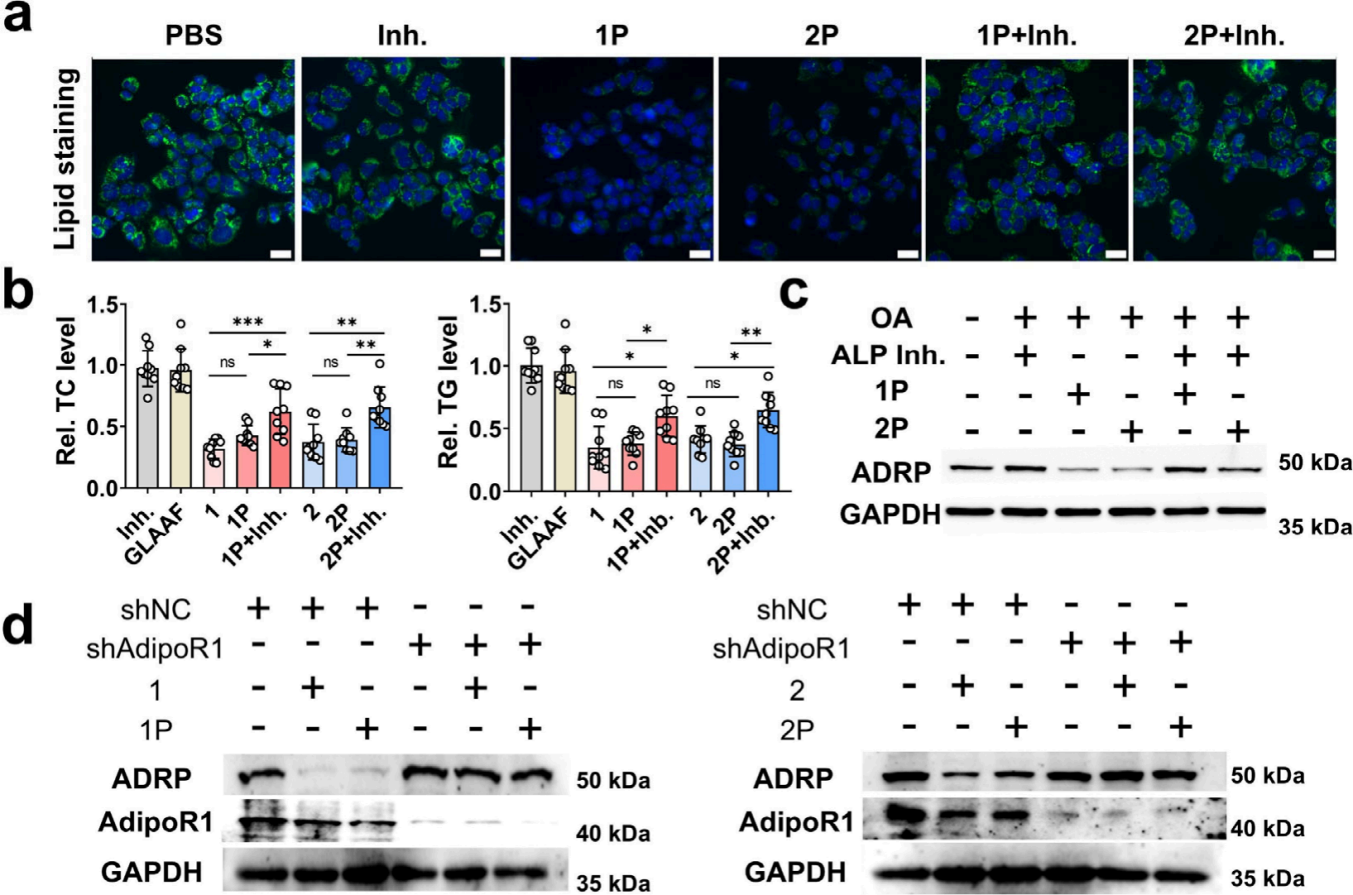
Enzyme-activated self-assembling peptides relieve lipid accumulation via AdipoR1. (a) Effect of ALP inhibition on peptide-mediated reduction of lipid accumulation in OA-induced Huh7 cells, visualized by BODIPY staining; scale bar 20 μm. (b) Quantification of total cholesterol (TC) and triglyceride (TG) levels after peptide treatment with or without ALP inhibitor (*n* = 9). (c) Western blot analysis of ADRP expression following peptide treatment in the presence or absence of ALP inhibitor. (d) Effect of peptides on ADRP expression in AdipoR1-knockdown Huh7 cells compared with the shNC-treated group. Mean ± SD; *p < 0.05; **p < 0.01; ***p < 0.001; ns, no significance.

To determine whether AdipoR1 is required for the activity of peptides, we evaluated ADRP expression in AdipoR1-knockdown Huh7 cells following peptides treatment. As shown in Figure 5d, neither **1P** nor **2P** was able to reduce ADRP levels in the absence of AdipoR1, underscoring the necessity of receptor engagement. Collectively, these findings confirm two critical steps in the peptide’s mechanism of action: (1) ALP-mediated dephosphorylation triggers self-assembly and activation of bioactivity; (2) AdipoR1 binding mediates downstream lipid metabolism regulation. This dual-step mechanism highlights the synergy between enzyme responsiveness and receptor targeting in driving the therapeutic efficacy.

### Enzyme-Activated Self-Assembling Peptides Improve Lipid and Glucose Homeostasis in NAFLD Models

To further validate the therapeutic efficacy of the self-assembling peptides *in vivo*, **1P** and **2P** were administered at a dosage of 20 mg/kg in high-fat diet (HFD)-induced NAFLD mice. The treatment schedule is illustrated in Figure 6a. Both peptides significantly modulated body weight, liver weight, and blood glucose levels after 8 weeks of treatment, indicating their systemic metabolic benefits (Figure 6a). To assess their lipid-lowering effects, serum and hepatic levels of TG and TC were measured. Both **1P** and **2P** substantially reduced lipid levels in the liver and bloodstream of NAFLD mice. Notably, **2P** exhibited therapeutic efficacy comparable to that of the positive control drug, liraglutide (Figure 6b). Oral glucose tolerance test (GTT) and subcutaneous insulin tolerance test (ITT) were conducted to assess the impact of **1P** and **2P** on insulin sensitivity and pancreatic islets function in HFD-mice. Area under the curve (AUC) analyses revealed that both peptides improved glucose metabolism, with **2P** nearly matching liraglutide in reversing HFD-induced insulin resistance (Figure 6c). Both **2P** and **1P** significantly reduced the LDL-c and fasting serum insulin levels, Figure S13. Additionally, Oil Red O staining of hepatic sections demonstrated a marked reduction in lipid droplets following peptide treatment, confirming their steatosis-alleviating effect (Figure 6d). Hematoxylin and eosin (H&E) staining revealed fewer vacuolated hepatocytes and reduced signs of adipose degeneration, suggesting mitigation of liver injury associated with lipid overload (Figure S14).

**Figure 6:**
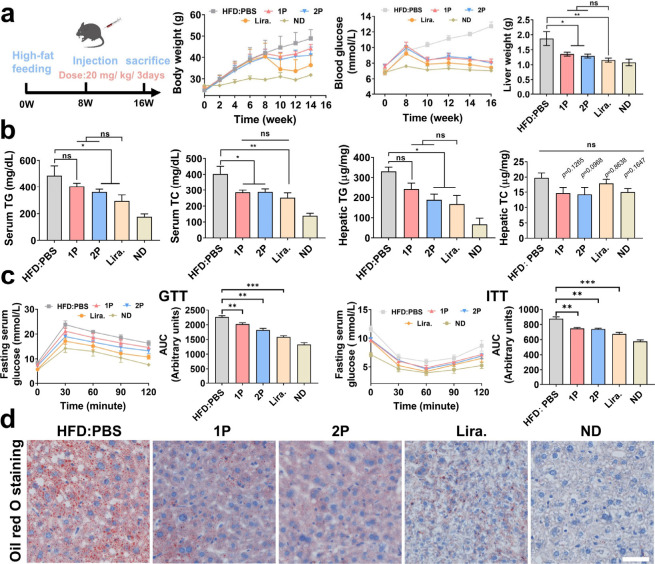
Therapeutic effects of self-assembling peptides in a NAFLD mouse model. (a) Schematic of NAFLD mouse model and treatment regimen, including tracking of body weight, blood glucose, and liver weight during administration (HFD: *n* = 7; 1P&2P: *n* = 9; Lira: *n* = 8; ND:*n* = 9). (b) Quantification of TG and TC levels in serum and liver tissue following treatment with **1P** and **2P**. (c) GTT and ITT conducted at week 16 to assess insulin sensitivity and pancreatic islets function, and AUC analyses for GTT and ITT shown on the right. (d) Hepatic pathology analyzed by Oil-red staining and H&E staining for evaluating lipid accumulation level; scale bar 50 μm. Mean ± SD; *p < 0.05; **p < 0.01; ***p < 0.001; ns, no significance.

Given the close association between NAFLD, inflammation, and hepatocellular injury, liver function markers were also evaluated. Alanine aminotransferase (ALT) and aspartate aminotransferase (AST) levels showed no significant differences compared with untreated controls, indicating that the peptides did not elicit additional hepatotoxicity (Figure S15). Histopathological analysis of major organs (heart, spleen, lung, and kidney) from peptide-treated mice revealed no visible abnormalities or lesions, supporting the in vivo safety of the adipo-peptides (Figure S16). Together, these results demonstrate that enzyme-activated self-assembling peptides effectively restore lipid and glucose homeostasis, reduce hepatic steatosis, and improve metabolic parameters in NAFLD mice—highlighting their potential as safe and effective therapeutics for metabolic liver disease.

### Self-Assembling Adipo-Peptides Enhance Metabolic Function and Weight Control in Diet-Induced Obese Mice

To evaluate the therapeutic efficacy of peptides in an obesity mouse model, mice were fed a 60 kcal% high-fat diet (HFD) for 12 weeks to induce obesity.^43^ Mice were then treated with 20 mg/kg of **1P** or **2P** every 3 days, with liraglutide used as a positive control (Figure S17a) As shown in Figure S17b,c, both peptides significantly suppressed weight gain and lowered blood glucose to near-normal levels after 12 weeks—achieving effects comparable to Liraglutide. ITT and GTT assays further confirmed that the metabolic benefits of peptide therapy (Figure S18a,b). Both **1P** and **2P** assess systemic energy metabolism, metabolic cage analysis was performed after 12 weeks of treatment. Peptide-treated mice. exhibited increased oxygen consumption, carbon dioxide production, energy expenditure, and respiratory exchange ratio (RER) during the dark cycle, suggesting enhanced utilization of both carbohydrates and lipids (Figure S19a–d). In summary, these findings demonstrate that self-assembling adiponectin-mimetic peptides can reduce weight gain, improve glycemic control and insulin responsiveness, and promote metabolic activity in obese mice.

### Transcriptomic Profiling Reveals Adipo-Peptide Regulation of Hepatic Metabolism and Inflammation in NAFLD Mice

To further elucidate the molecular mechanisms underlying the therapeutic effects of Adipo-peptides, we conducted bulk RNA sequencing on liver tissues collected from NAFLD mice following 8 weeks of **2P** treatment (Figure 7a). Interestingly, only 106 differentially expressed genes (DEGs; |FC ≥ 1.5| and FDR = 0.05) were identified when comparing the **2P**-treated group to the control, indicating a highly specific transcriptomic response mediated through AdipoR1 signaling. Gene Ontology (GO) analysis via Metascape revealed that the upregulated genes were predominantly involved in lipid and glucose metabolism, metabolic regulation, inflammation response, antioxidant activity, and the MAPK signaling pathway (Figure 7b,c). These enriched biological processes align well with the observed improvements in glucose-lipid metabolism seen in both cellular and in vivo models. Gene set enrichment analysis (GSEA) further confirmed that adipogenesis, biosynthesis of unsaturated fatty acids, and MTORC1 signaling upregulated in HFD-fed mice, but were markedly downregulated following **2P** treatment (Figure 7d). These transcriptomic shifts suggest that the peptide reverses key pathological processes in NAFLD.

**Figure 7:**
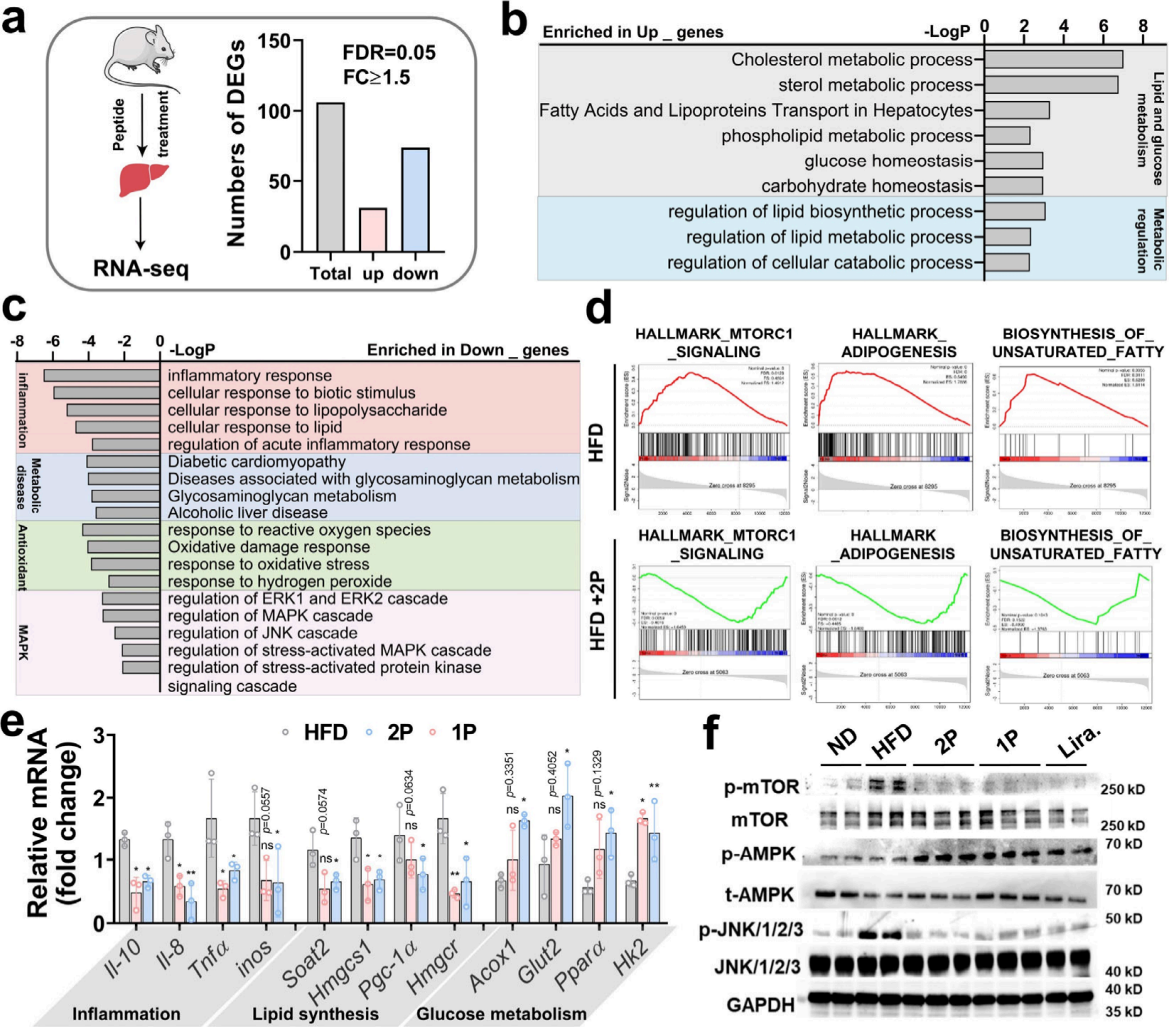
Transcriptomic and molecular effects of 2P treatment in hepatic tissue of NAFLD mice. (a) Schematic of RNA-seq workflow (left) and total DEGs identified between **2P**-treated and control groups (right). (b, c) GO analysis of upregulated and downregulated DEGs in liver tissues following **2P** treatment. (d) GSEA of selected metabolic and signaling pathways in **2P** versus vehicle-treated NAFLD mice. (e) qRT-PCR validation of selected genes involved in lipid metabolism, glucose regulation, and inflammation. (f) WB analysis of AMPK, JNK, and mTOR signaling pathways in hepatic tissue after peptide treatment. Data are presented as Mean ± SD; *p < 0.05; **p < 0.01; ***p < 0.001; ns, no significance.

qRT-PCR validation confirmed the RNA-seq findings. Treatment with **2P** led to the downregulation of lipid synthesis genes (*Soat2*, *Hmgcs1*, *Pgc-1α*, and *Hmgcr*) and pro-inflammatory cytokines (*Il-10*, *Il-8*, *Tnfα*, *Inos*), while genes associated with lipid oxidation and glucose metabolism (*Acox1*, *Glut2*, *Pparα*, and *Hk2*) were significantly upregulated (Figure 7e).^44,45^ These molecular changes reinforce the role of Adipo-peptides in modulating hepatic lipid metabolism and inflammation. To confirm that these transcriptomic changes are driven by activation of the adiponectin receptor pathway, we examined downstream signaling proteins using Western blot analysis. As illustrated in Figure 7f, **2P** treatment activated AMPK and inhibited mTOR signaling, aligning with its function in promoting lipid oxidation and suppressing lipid synthesis.^46,47^ Additionally, the JNK pathway was deactivated following peptide administration, corroborating its enrichment observed in the GO analysis. Collectively, these data demonstrate that the therapeutic effects of self-assembling Adipo-peptides in NAFLD are mediated through precise transcriptomic reprogramming, involving activation of AdipoR1-dependent metabolic and anti-inflammatory pathways.

## Conclusion

Peptide-based therapeutics represent a rapidly expanding class of agents for diverse diseases, owing to their favorable safety profiles and molecular specificity. This has spurred increasing interest in developing peptide drugs for metabolic liver diseases such as NAFLD and NASH. However, challenges including limited stability and the lack of controlled, site-specific activation have restricted their clinical translation.^48^ Therefore, it is necessary to develop new peptide therapeutics for NAFLD that combine enhanced stability with efficient, targeted activation.

In this study, we present a supramolecular approach to recapitulate the multimeric structure and function of adiponectin using enzyme-activated self-assembling peptides. By rationally designing two ALP-activatable sequences, **1P** and **2P**, we achieved in situ formation of nanofibers that mimic the high-molecular-weight (HMW) forms of adiponectin and effectively engage AdipoR1. These assemblies exhibited superior physicochemical stability, bioactivity, and therapeutic efficacy in both cellular and murine models of nonalcoholic fatty liver disease (NAFLD). Mechanistically, the adipo-peptides alleviated lipid accumulation, inflammation, and oxidative stress, while also improving glucose homeostasis. Transcriptomic and biochemical analyses confirmed modulation of metabolic and inflammatory pathways, including AMPK activation and mTOR suppression, supporting their role as functional adiponectin mimetics.

Despite these advances, several limitations remain. First, while we demonstrate AdipoR1-dependency and favorable therapeutic outcomes, detailed mechanistic insights into intracellular signaling cascades and long-term outcomes require further investigation. Second, the systemic metabolic benefits observed here suggest that these peptides may hold promise beyond NAFLD, particularly in obesity and type 2 diabetes,^49,50^ these potential merits comprehensive evaluation. Finally, optimization of therapeutic efficacy and validation in larger animal models will be essential steps toward translation. Relatively high injection doses may pose risks for practical applications. Strategies to reduce the effective concentration of peptides warrant further investigation, including enhancing target specificity and improving bioavailability.^51,52^ For example, AI-assisted peptide design could increase receptor selectivity, while liver-targeted delivery systems could enhance tissue-specific accumulation and overall efficacy.^53,54^ Overall, this work establishes a versatile platform for constructing bioactive, enzyme-triggered self-assembling peptides that emulate multimeric proteins. By integrating structural biomimicry with targeted activation, our strategy provides a promising path toward next-generation nanotherapeutics with broad translational relevance in metabolic disease.^7,55,56^

## Supplementary Material



## Notes

Materials and methods, synthesis, and characterization of all peptides (PDF)

## Notes

J.S., Z.L., D.Y., Q.J, and Z.Z. are inventors on pending patent applications (No. 202510569247.3).

## Abbreviations

NAFLD: nonalcoholic fatty liver disease
ALP: alkaline phosphatase
HFD: high-fat diet
NASH: nonalcoholic steatohepatitis
PPAR: peroxisome proliferator-activated receptor
FXR: farnesoid X receptor
TEM: transmission electron microscopy
GPC: gel permeation chromatography
TG: intracellular triglyceride
TC: total cholesterol
OA: oleic acid
GTT: glucose tolerance test
ITT: insulin tolerance test
