# Supplementary material for: Enzyme-Activated Self-Assembling Peptides Mimicking Adiponectin Multimers for Nonalcoholic Fatty Liver Disease Therapy
Source: ACS Cent Sci. 2026 Feb 11;12(2):243–53. doi: 10.1021/acscentsci.5c02405 (PMC12947284; doi:10.1021/acscentsci.5c02405)
Supplement: Supplementary file 1 [file oc5c02405_si_001.pdf]

Supporting Information

of

Enzyme-Activated Self-Assembling Peptides Mimicking Adiponectin Multimers for Nonalcoholic Fatty Liver Disease Therapy

Zenghui Li,[1†] Shuangdi Duan,[2†] Zihao Zhu,[1] Hong Han,[1] Nong Qin,[1] Qiaoqiao Ji,[1] Dan Yuan,[1*] Junfeng Shi[1,3*]

[1] Hunan Provincial Key Laboratory of Animal Models and Molecular Medicine, State Key Laboratory of Chemo/Bio-Sensing and Chemometrics, School of Biomedical Sciences, Hunan University Changsha, Hunan 410082, China.

[2] The First Affiliated Hospital of Guangxi Medical University, Guangxi Medical University, Nanning 530021, Guangxi, China.

[3] Shenzhen Research Institute, Hunan University, Shenzhen 518000, Guangdong Province, China.

* Corresponding Authors: Yuandan@hnu.edu.cn, Jeff-Shi@hnu.edu.cn.

Table of Content

1. Materials and synthesis methods S3

1.1 Materials S3

1.2 Peptide synthesis S3

2. Physicochemical characterization S3

2.1 Transmission electron microscopy (TEM) S3

2.2 Gel permeation chromatography (GPC) S4

2.3 Critical aggregation concentration (CAC) measurement S4

2.4 Microscale thermophoresis (MST) assay S4

2.5 Serum stability and hemolysis studies S5

3. Cell experiments S6

3.1 Cell viability assay S6

3.2 Plasmid transfection S6

3.3 Western blotting (WB) S7

3.4 Quantitative Real-Time PCR (qRT-PCR) S7

3.5 Laser scanning confocal microscope S8

3.6 Immunofluorescence staining S8

3.7 ROS level detection S9

3.8 Pull-down assay of biotin-labeled peptides S9

3.9 Lipid quantification S10

4. Animal experiment S10

4.1 Peptide safety and therapeutic effect study in mouse models S10

4.2 Therapeutic effect S11

5. Supplementary figures S14

6. Supplementary tables S23

1. Materials and synthesis methods

1.1 Materials

Rink Amide (AM) Resin, Fmoc-protected amino acids and O-Benzotriazole-N, N, N', N'-tetramethyl-uronium-hexafluoro-phosphate (HBTU) were purchased from GL Biochem (Shanghai) i Co., Ltd. Trifluoroacetic acid (TFA), acetonitrile, methanol, hexane, piperidine, dimethylformamide were purchased from Yi nuo kai. All antibody were commercially available, GAPDH and Secondary antibody were purchased from Proteintech group and Zen-bioscience. AdipoR1 were obtained from Beyotime Biotechnology group. The mTOR and p-mTOR, p-AMPK, t-AMPK were purchased from CST Group. JNK1/2/3 and p-JNK1/2/3 were provided from Selleck group (Selleckchem.com). All the solvents and reagents were used directly as received from commercial sources without further purification.

1.2 Peptide synthesis

All peptides were synthesized by standard Fmoc solid-phase peptide synthesis on a CSBio 136S peptide synthesizer, with Rink Amide AM Resin and activation by HCTU. After the synthesis of the peptide chain, the resin should be washed successively with DMF, DCM, MEOH and n-hexane, Finally, the peptide chain without side chain protecting groups was cleaved from the resin with TFA/thioanisole/H2O (95:2.5:2.5) for 3 h under nitrogen atmosphere. Crude peptide was obtained by concentrating the filtrate and precipitating it with cold ether. The crude product was purified by reversed phase high performance liquid chromatography (RP-HPLC) and lyophilized to obtain dry powder. All purified peptides were analyzed using analytical HPLC (LC-20AT, Shimadzu, Japan) and MALDI-TOF MS (UltrafleXtreme, Bruker).

2. Physicochemical characterization

2.1 Transmission electron microscopy (TEM)

The sample was prepared by diluting 5 μL of a 1.0 wt% peptide working solution in an appropriate volume of ddH_2O. A drop of this solution was placed on a 200-mesh copper grid coated with carbon film (#BZ11032b, EMCN) and left to stand for 1 min before blotting with filter paper. Subsequently, 2% uranyl acetate was applied for 1-2 min, blotted again, and left to air dry. Images were captured using a Hitachi 7650 transmission electron microscope at an accelerating voltage of 80 kV.

2.2 Gel permeation chromatography (GPC)

The molecular weights (MW) range of the peptide assembles were determined by gel permeation chromatography (PL-GPC50, Agilent) equipped with a refractive index detector. Measurements were performed at room temperature using PBS as the mobile phase at a flow rate of 1.0 mL min^{-1}. Molecular weights were calculated using the universal calibration method with polystyrene standards (PEG/PEO).

2.3 Critical aggregation concentration (CAC) measurement

The critical aggregation concentration (CAC) was determined by measuring the fluorescence of 8-anilino-1-naphthalenesulfonic acid (ANS) incubated with varying peptide concentrations. Samples were mixed with an equal volume of 2×PBS buffer at 37°C for 1 h. After incubation with 20 μM ANS, fluorescence spectra were recorded on a F-7000 spectrofluorometer (Hitachi, Japan) from 400 to 580 nm (λex = 340 nm) to determine the CAC.

2.4 Microscale thermophoresis (MST) assay

All measurements were conducted using the Monolith NT 115 instrument. Each measurement utilized a total volume of 10 μL loaded into standard capillaries. The ligand peptide was prepared by diluting it into a series of concentrations from a 5 mM stock solution. Prior to the measurements, the purified AdipiR1 recombinant protein (UAPA147Hu02, Wuhan CLOUD-CLONE CORP.) was

diluted in assay buffer and labeled using the MO RED-tris-NTA Protein Labeling Kit (#MO-L018, NanoTemper). After a 30 min incubation period, the peptide was added to achieve final concentrations of 50 nM for dye-labeled AdipiR1 and a range of concentrations for the peptide stock. Following an additional 30 min incubation, samples were loaded into mini capillaries (#914941, HIRSCHMANN) and analyzed using a Monolith NT.115 instrument. Measurements were performed immediately using the red channel with 40% excitation power and medium MST power. Data were analyzed with MO Affinity Analysis software (v2.3).

2.5 Serum stability and hemolysis studies

Stability, Peptide were dissolved in 1:3 serum: buffer (20 mM Tris-HCl, 100 mM NaCl, pH 7.4) to a final concentration of 200 μM. Samples were then incubated at 37 °C in an incubator. At 0, 0.5, 1, 2, 4 or 8 h of incubation a 100 μL aliquot was removed and diluted with an equal volume of 15 wt% Trichloroacetic acid (TCA) in ddH_2O, and the sample was kept on ice for 15 min. Samples were then centrifuged at 13,000 rpm for 10 min, and supernatant collected and subjected to analytical HPLC.

Hemolysis investigations were conducted by procuring freshly drawn blood from healthy human volunteers into heparinized tubes, followed by subjecting it to centrifugation at a speed of 3,000 rpm for a duration of 10 min at a temperature of 4 °C. Red blood cells (RBCs) were subjected to three washes with a hemolysis buffer composed of 10mM Tris, 150 mM NaCl, pH 7.4. Subsequently, a solution of RBCs in the hemolysis buffer, with a concentration of 0.25% v/v, was carefully prepared. In a 96-well plate, a volume of 75 μL of the RBC solution was admixed with an equivalent amount of a 2× peptide solution, previously dissolved in the hemolysis buffer, to initiate the assay. Negative and positive controls were established using a blank and a buffer containing 1% Triton-X100, respectively. The samples were subjected to

incubation for a duration of 24 h, accompanied by gentle agitation. Subsequently, the plates were centrifuged at 4,000 rpm for 10 min at a temperature of 4 °C to precipitate intact RBCs. Following this, a volume of 100 μL of the supernatant from each well was extracted and transferred to an empty 96-well plate. The absorbance was then measured at a wavelength of 415 nm utilizing a microplate reader.

3. Cell experiments

3.1 Cell Viability Assay

Cells were seeded in 96 plates at a density of 8,000/well and continued to culture overnight at incubator (37 °C, 5% CO_2). Peptide stock solutions were prepared in ddH_2O and subsequently diluted with DMEM to obtain working solutions at the desired concentrations. Cells were incubated with peptide work solution for 24 h. Then the 100 μL 10 %v/v MTT (M1020, Beijing Solarbio Science & Technology Co., Ltd.) solution was added to the plate well for 3 h. After incubation, the plates were subjected to the UV plate reader (SpectraMax M2, US Molecular Devices) to record 450 nm data. The absorbance of the negative controls was subtracted from each sample as a blank, and the percent viability was calculated as follows: (Absorbance peptide-treated cells / Absorbance untreated cells) ×100. GraphPad Prism 7.0 software was used to process data and draw graphs.

3.2 Plasmid transfection

All plasmids (**Table S1**) were sequenced and validated by Beijing Tsingke Biotechnology Co. Ltd. Huh7 cells seeded in a 6-well plate were transfected with shNC (vector) or shAdipoR1 (1-4) using PEI (#23966, Polysciences) according to the following procedure: Huh7 cells were refreshed with 1.5 mL DMEM containing 10% FBS (without penicillin–streptomycin) and incubated for 12 h prior to subsequent experiments. For transfection (mass ratio, PEI :

shRNA=3:1), solution A was prepared by mixing 10.8 µL PEI (1 mg/mL) with 140 µL Opti-MEM (#31985-070, Gibco) and incubated for 5 min at room temperature, while solution B was prepared by combining the shRNA plasmid in 140 µL Opti-MEM and incubated for 5 min. Solutions A and B were then combined, mixed thoroughly, and incubated for at least 30 min at room temperature before being added dropwise to the cells. After 6 h, the medium was replaced with 1.5 mL fresh DMEM (10% FBS, without penicillin–streptomycin). 48 h post-transfection, the cells were collected and centrifuged at 1,000 rpm for 4 min for following study (CLSM or WB)

3.3 Western blotting (WB)

Cells were seeded into a 6-well plate at a density of $3x10^5$ cells per well and cultured for 24 h. Following treatment with the 50, 100, 200 µM peptide or ALP inhibitor for the appropriate duration, the treated cells were lysed using 1x passive buffer supplemented with protease inhibitors such as PMSF (#S3025, Selleck) and a phosphatase cocktail (K1015, APE×BIO). The concentration of total protein in the lysates was determined using Quick Start Bradford 1x Dye Reagent (#5000205, Bio-Rad), and the protein concentration quantified by measuring absorbance at 595 nm. Subsequently, the protein samples were mixed with 1×Loading buffer (Cat No. #LT101S; Epizyme, Shanghai) and denatured by heating them at 95-100°c for 5 min and subjected to the Western blotting assay.

3.4 Quantitative Real-Time PCR (qRT-PCR)

The primer sequences for all genes are listed in Supplementary **Table S2** and were synthesized by Tsingke Biotechnology Co., Ltd. Total RNA was extracted using the RNA-easy Isolation Reagent (R701-01, Vazyme), and RNA concentration was determined with a NanoDrop (1011U, NanoDrop, USA). One microgram of total RNA was placed into PCR tube (BIOLAND, Hangzhou, china) and reverse-transcribed into

cDNA using HiScript Ⅲ RT SuperMix for qPCR (+gDNA wiper, R323-01, Vazyme). Quantitative PCR was performed with ChamQ Universal SYBR qPCR Master Mix (Q711-02, Vazyme). Relative mRNA expression levels of genes involved in lipid synthesis (*Soat2, Hmgcs1, Pgc-1α, and Hmgcr*), pro-inflammatory cytokines (*Il-10, Il-8, Tnfα, and Inos*), lipid oxidation, and glucose metabolism (*Acox1, Glut2, Pparα*, and *Hk2*) were normalized to the control *Actin* using the 2^−ΔΔCt method. All experiments were independently repeated at least three times.

3.5 Laser scanning confocal microscope

Peptide uptake, Cells were seeded on 35 mm confocal dishes (#D35-20-1-N, Cellvis.) at a density of $5x10^4$/well and continued to culture for 24 h in a 37 °C incubator with 5% CO_2. Then, the cells were washed twice with PBS buffer and incubated with 1 mL peptide work solution at 37 °C with 5 % CO_2 for desired time. The incubated solution of cells was removed, and the following was washed twice with PBS. Then the cells were stained with 2 μg/mL Hoechst 33342 (#H1399, Thermo Fisher) for 15 min, cell imaging was performed on a Zeiss-LSM980 microscopy with 63×oil objective, and the cells were maintained in a cell living imaging buffer during confocal imaging.

3.6 Immunofluorescence staining

Huh7 cells were seed on round coverslip (#BS-18-RC, Bio-sharp) in 12-well plates. After cell adhesion, cells were incubated with the FITC labeled peptide and fixed with 4% paraformaldehyde. Permeabilization was performed with 0.3% Triton X-100, followed by blocking with 5% goat serum in PBST (PBS containing 0.1% Tween 20). Coverslips were then incubated with the primary antibody (anti-AdipoR1, #AF2131, Beyotime, 1:500) diluted in PBST supplemented with 5% goat serum at 4 °C overnight. After three times PBST washes, the samples were incubated with CoraLite594-conjugated secondary antibody (#SA00013-4, Proteintech) for 1 h at room temperature. Cells were

washed again with PBST and stained with Hoechst 33342 for 15 min. Immunofluorescence images were captured using a Zeiss-LSM980 microscopy. For co-localization analysis, merged pixels from the green (peptides) and red (AdipoR1) channels were masked to detect co-localized regions using the CoLoc. module of ZEN 3.9 software.

3.7 ROS level detection

Cells were seeded at a density of 1×10^5 cells/well on confocal dishes and incubated overnight to allow adherence. Cells were then treated with 400 μM oleic acid (OA) for 24 h to induce lipid accumulation. Following the indicated peptide treatments, the medium was removed and cells were washed three times with PBS. Cells were subsequently stained with CellROX Green (#C10444, Invitrogen) for 20 min, followed by Hoechst 33342 for nuclear staining. Confocal microscopy was used for imaging, whereas flow cytometry (CytoFLEX, Beckman Coulter) was performed for quantitative analysis (nuclear staining was omitted for flow cytometry).

3.8 Pull-down assay of biotin-labeled peptides

Cell pellets were lysed in Lysis Buffer (50 mM HEPES, 150 mM NaCl, 1 mM EDTA, 0.1% NP40, pH 7.5) supplemented with 1x Protease Inhibitor Cocktail (Selleck, #B14001) and Phosphatase Inhibitor Cocktail, and the protein was harvested and quantified using BCA kits. The protein was then incubated with biotinylated peptide in Pulldown Buffer (50 mM Tris, 0.1% NP40, 150 mM NaCl, pH 7.5) at 4°C with rotation. After 4 h, 20 μL of streptavidin magnetic beads (#L-1012, Biolinkedin), previously blocked with Pulldown Buffer supplemented with 0.5% BSA, were added to biotinylated peptide pulldown and allowed to interact with the protein-peptide mixture overnight at 4 °C with rotation. Following incubation, the beads were washed 3 times with Pulldown Buffer using a magnetic separation rack. After washing, the supernatant was removed, and the bound protein was eluted by adding 50 μL of 1x Loading Buffer and

boiling the samples for 10 min at 95°C before loading into a gel for further WB analysis.

3.9 Lipid quantification

Cells were trypsin digested into suspension and counted. They were seeded at a density of 1×10^5 cells per well in 24-well plates or on confocal dishes and incubated overnight to allow adherence. Subsequently, cells were incubated with 400 μM oleic acid (OA) for 24 h to induce lipid accumulation. After treatment with the peptides for an additional 24 h, the medium was removed, cells were washed three times with PBS, and stained with BODIPY 493/503 (#D3922, Invitrogen) for 20 min, followed by Hoechst3342 for nuclear staining. Confocal microscopy was then used for observation and quantitative analysis. BODIPY staining is widely used to visualize intracellular neutral lipid droplets. For quantitative determination of total cholesterol (TC) and triglycerides (TG), 100 μL of lipid extraction solution (hexane / isopropanol, 3:2 v/v) was added per well. The plate was gently tilted and repeatedly pipetted to ensure complete coverage of the cells by the extraction solution. The extract was collected into 1.5 mL centrifuge tubes and evaporated on a 60 °C metal bath to remove all organic solvents. Lipids were then resuspended in 0.1% Triton X-100 by vortexing and incubating for 30 min to ensure full solubilization. Concurrently, 200 μL of 0.2 mM NaOH was added per well on the plate, which was shaken at room temperature for 3 h to extract total cellular protein. Finally, 10 μL of the extract was used to quantify TC and TG levels using the GPO–PAP method in accordance with the TC and TG colorimetric kit instructions (Nanjing Jiancheng Bioengineering Institute, TC: #A111-1-1; TG: #A110-1-1), and the results were normalized to the corresponding cellular protein concentrations.

4. Animal experiment

4.1 Peptide safety and therapeutic effect study in mouse models

All animal research conducted in this study was approved by the Ethics Committee for Animal Experiments at HNU University (Approval No. HNU-IACUC-2021-102).

4.2 Therapeutic effect

Six-week-old C57BL/6J male mice were obtained from GemPharmatech LLC. (Jiangsu, China). After a one-week acclimation, mice were fed a 60 kcal % high-fat diet (XTHF60, Xietong Pharmaceutical Bio-Engineering) for 8 weeks to induce hepatic steatosis and establish a non-alcoholic fatty liver disease (NAFLD) model. For the obesity model, mice continued the same diet for 12 weeks. Prior to peptide treatment, mice were randomized into groups based on their body weight. Peptides (1P and 2P) were freshly prepared before each experiment. Briefly, the peptides were dissolved in ddH_2O, followed by the gradual addition of 0.1 M NaOH until the solution became clear. To adjust the pH to approximately 8, an equal volume of 2× PBS (pH 7.4; 274 mM NaCl, 5.4 mM KCl, 20 mM Na_2HPO_4, 3.6 mM K_2HPO_4) was added. Mice received intraperitoneal injections of the peptides (20 mg/kg, 200 µL) every three days for eight weeks. Liraglutide (200 µg/kg, 200 µL) was used as a positive control, PBS as a vehicle negative control, and a normal diet–fed group was included as a reference. During the treatment period, body weight and blood glucose levels were monitored every two weeks. After treatment, insulin tolerance tests (ITT) and glucose tolerance tests (GTT) were performed. Mice were subsequently sacrificed, and tissues were collected for immunohistochemistry, while blood was harvested for biochemical assays.

***GTT*：** Mice were fasted for 12 h (access to ddH_2O) before the test. Glucose dosage (1.5 g/kg body weight) was prepared as a 20% (w/v) solution and administered intraperitoneally. Baseline blood glucose was measured via tail blood at 0 min. Further measurements were taken at 30-, 60-, 90-, and 120-min

post-injection. Glucose values were plotted and the area under the curve (AUC) calculated to evaluate glucose tolerance.

ITT : Mice were fasted for 4–6 h before testing. Insulin (1.2 mU/g body weight) was diluted appropriately and administered intraperitoneally. Baseline glucose was measured at 0 min (tail blood) using the blood glucose meter Yuwell 660, followed by measurements at 30-, 60-, 90-, and 120-min. Decreases in blood glucose were recorded and used to assess insulin sensitivity.

Blood sample processing: Blood was collected into 1.5 mL anticoagulant tubes and allowed to stand at room temperature for 30 min, then centrifuged at 3,000 rpm for 10 min. Serum was transferred to fresh tubes and stored at −20 °C. Serum was used to quantify TG, TC. Additionally, ALT and AST levels were measured using the Alanine Aminotransferase Assay Kit (#C009-2-1) and Aspartate Aminotransferase Assay Kit (#C010-2-1), Ldl-c respectively, both purchased from Nanjing Jiancheng Bioengineering Institute.

Liver tissue processing: Approximately 0.1 g of liver tissue was placed in a 1.5 mL tube with 1 mL PBS and grinding beads, then homogenized at 80 Hz for 120 s. Samples were centrifuged at 12,000 rpm for 15 min, and the supernatant was aliquoted and stored at −80 °C. Tissue homogenates were diluted 100–200× for protein quantification and used for TG/TC assays and Western blot. For Western blot, samples were normalized by protein concentration before electrophoresis, membrane transfer, and antibody detection to assess target protein expression.

***Hematoxylin and eosin (H&E) and* Oil Red O staining:** At the conclusion of the study, mice were euthanized, the major organ tissues were harvested for histological evaluation. The collected samples were fixed, dehydrated, paraffin-embedded, and sectioned into 4 μm-thick slices. For H&E staining, tissue sections were processed using standard protocols and imaged with a 3DHISTECH slide scanner (Budapest, Hungary). Oil Red O staining as following steps, frozen tissue sections were prepared, air-dried, and fixed in 10% neutral formalin for 10 min, followed by rinsing with distilled

ddH_2O. Sections were then stained with freshly prepared Oil Red O working solution for 10-15 min, washed gently with ddH_2O, counterstained with hematoxylin, and imaged with a slide scanner.

5. Supplementary figures

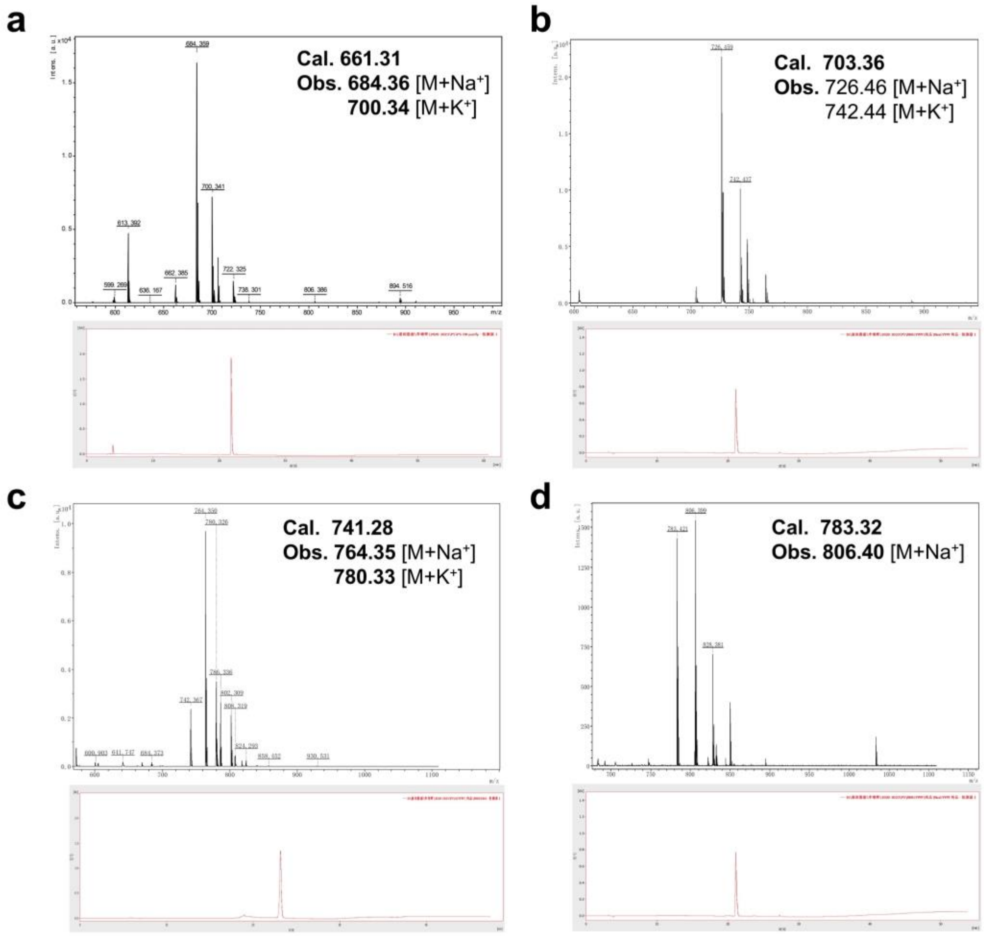

Figure S1. Analytic HPLC and mass spectra of all peptides studied. **(a) 1**, **(b) 2**, **(c) 1P**, **(d) 2P** respective shown in this figure.

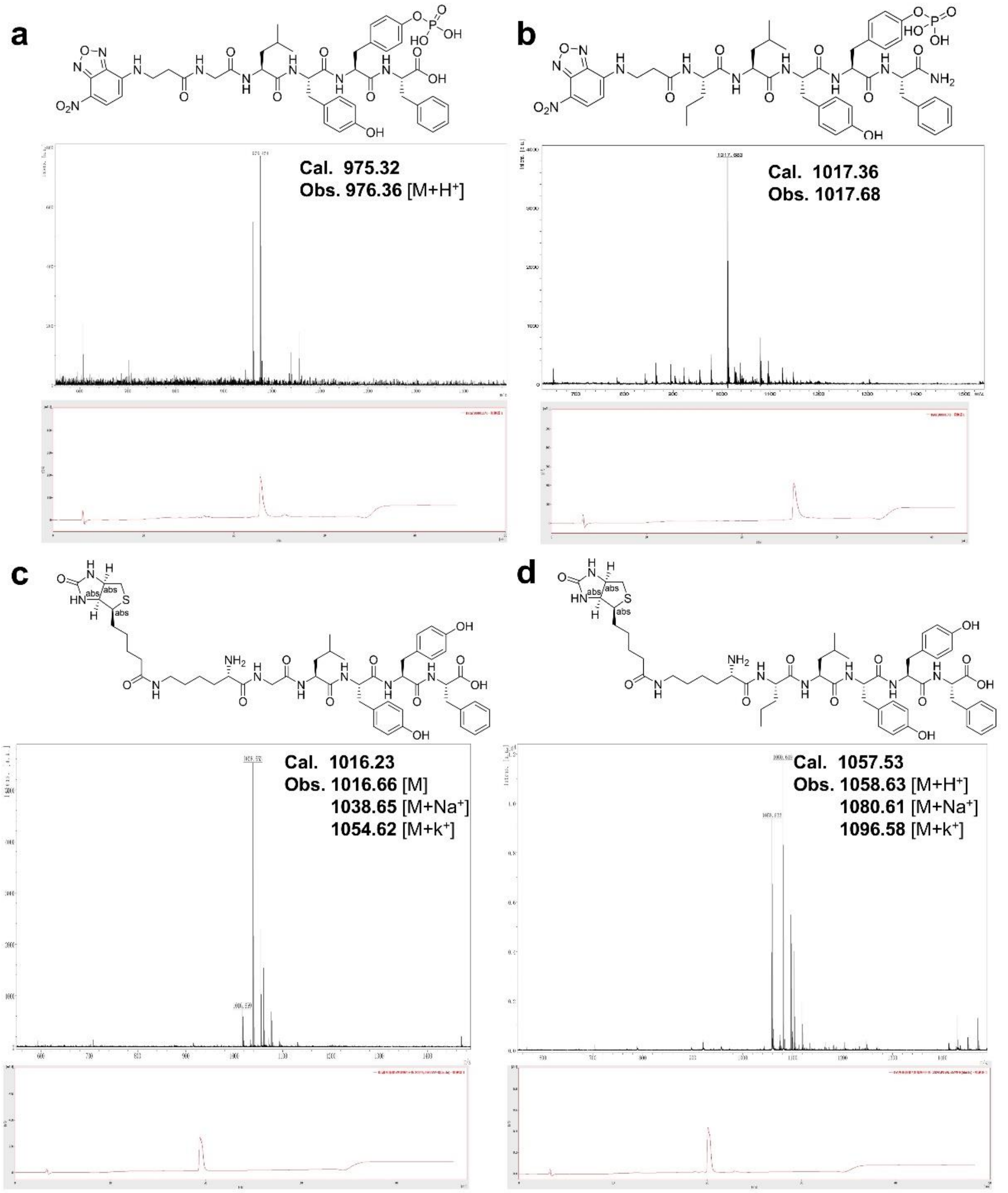

Figure S2. Analytic HPLC and mass spectra of all peptides studied. **(a) NBD-1P**, **(b) NBD-2P**, **(c) Biotin-1**, **(d) Biotin-2** respective shown in this figure.

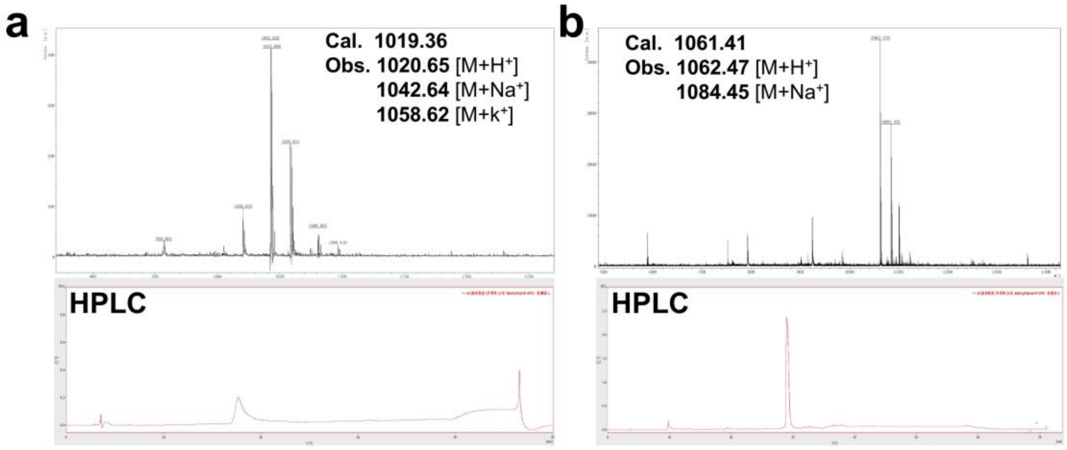

Figure S3. Analytic HPLC and mass spectra of all peptides studied. **(a) FITC-1**, **(b) FITC-2** respective shown in this figure.

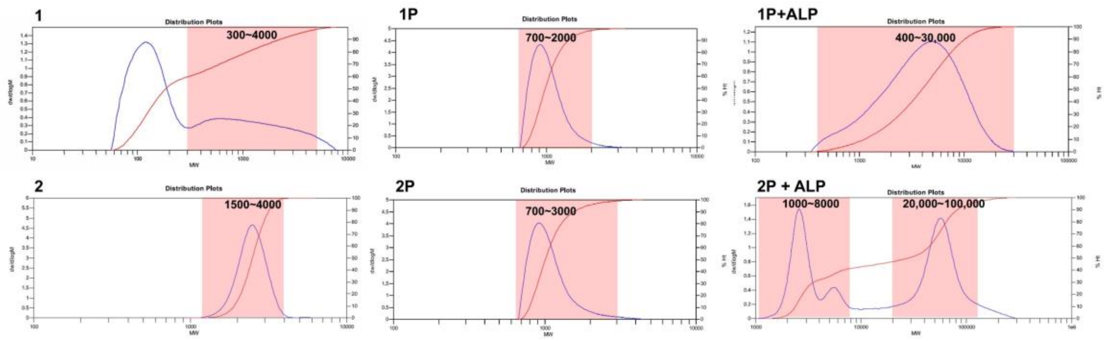

Figure S4. Gel Permeation Chromatography (GPC) spectrum of the peptide solution (**1, 1P** and **2, 2P**) and the peptide assemblies generated by phosphorylated peptides (**1P** and **2P**) incubated with ALP.

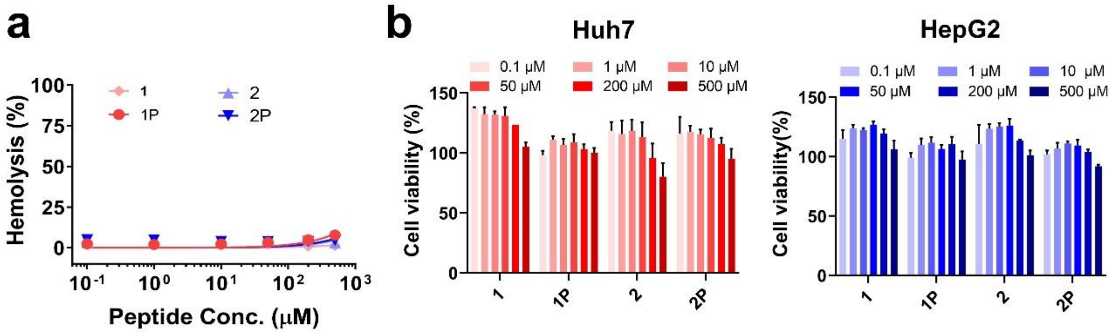

Figure S5. **(a)** Cytotoxicity of adipo-derived peptide towards Huh7 cell and HepG2 cell in 24 h. **(b)** Hemolysis test with primary human blood cells evaluating the biological safety of **1** and **1P**, **2**, **2P**.

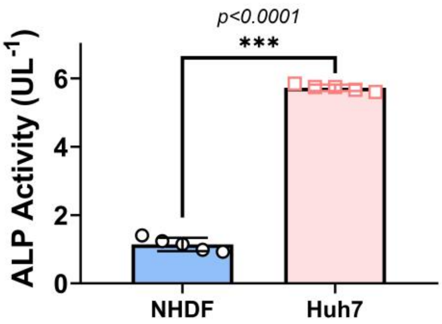

Figure S6. Quantification of the ALP activity of various cell lines. Mean ± SD. *p < 0.05; **p < 0.01; ***p < 0.001; ns, no significance.

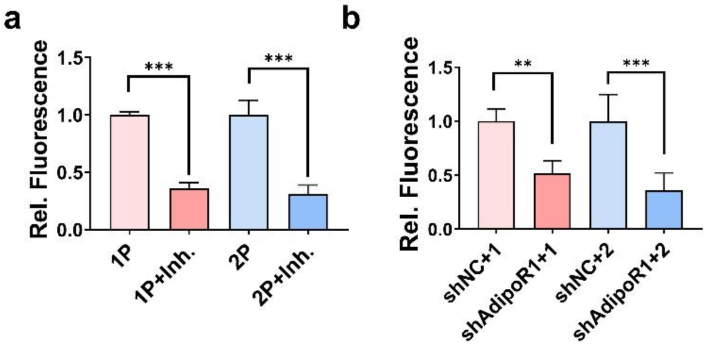

Figure S7. Flow cytometer study enzyme-activated peptide self-assembly and AdipoR1 binding. (a) Flow cytometry analysis (n = 4) of Huh7 cells incubated with 100 μM NBD-labeled **1P** or **2P** for 3 h in the presence or absence of the ALP inhibitor levamisole (1 mM), demonstrating ALP-dependent peptide self-assembly. (b) Flow cytometry analysis (n = 4) of Huh7 cells transfected with either control shNC or AdipoR1-targeting shRNA (shAdipoR1) after incubation with 50 μM FITC-labeled **1** or **2**, confirming receptor-dependent peptide binding. Data are presented as mean ± SD. *p < 0.05; **p < 0.01; ***p < 0.001; ns, not significant.

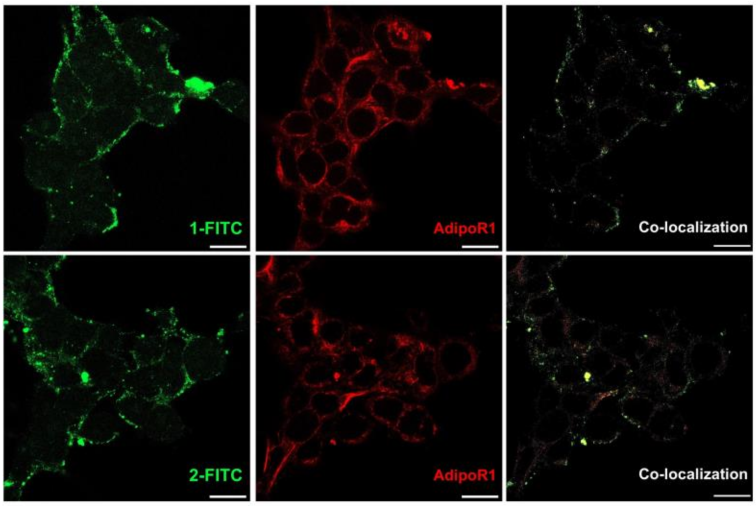

Figure S8. Co-localization analysis of peptides **1** and **2** (green channel) with AdipoR1 (red channel) was performed using ZEN 3.9 software by masking merged pixels, scale bar 20 μm.

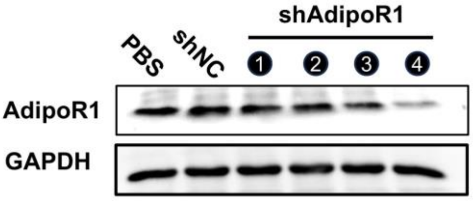

Figure S9. Western blot analysis showing AdipoR1 expression in huh7 cell lines transduced with shNC or AdipoR1-specific hairpin shRNA1-4.

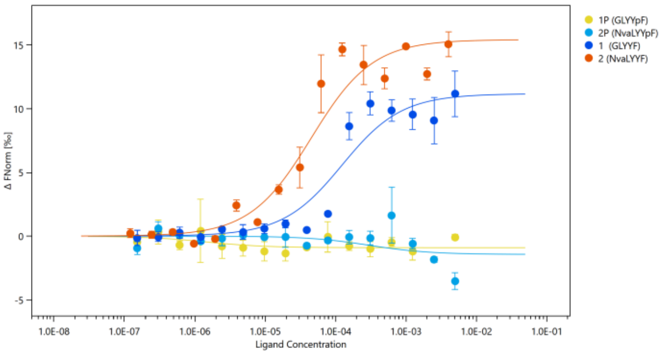

Figure S10. Microscale thermophoresis (MST) assays measuring the binding affinity between peptide **1**, **2**, **1P**, **2P** and AdipoR1 recombinant protein.

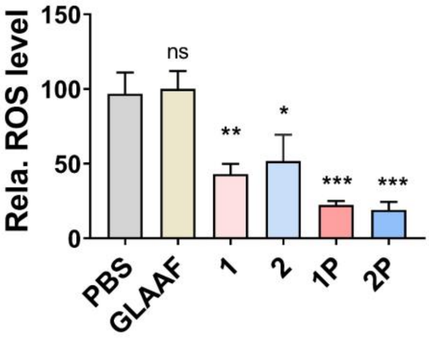

Figure S11. Flow cytometry analysis (n=3) of ROS levels measured by fluorescence staining following peptide treatment. Data are presented as mean SD. *p < 0.05; **p < 0.01; ***p < 0.001; ns, not significant.

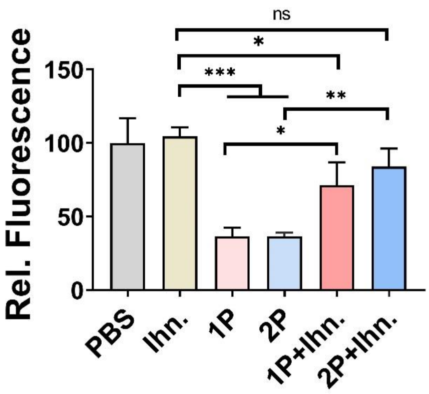

Figure S12. Flow cytometer analysis (n=3) of lipid droplets accumulation in OA-treated Huh7 cells following peptide (**1P**, **2P**) treatment with or without ALP inhibitor. Mean ± SD. *p < 0.05; **p < 0.01; ***p < 0.001; ns, no significance.

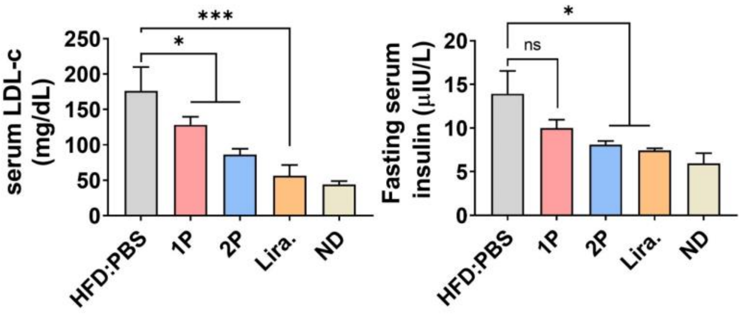

Figure S13. Quantification of LDL-C and insulin in NAFLD mouse after **1P** and **2P** treatment. Mean ± SD. *p < 0.05; **p < 0.01; ***p < 0.001; ns, no significance.

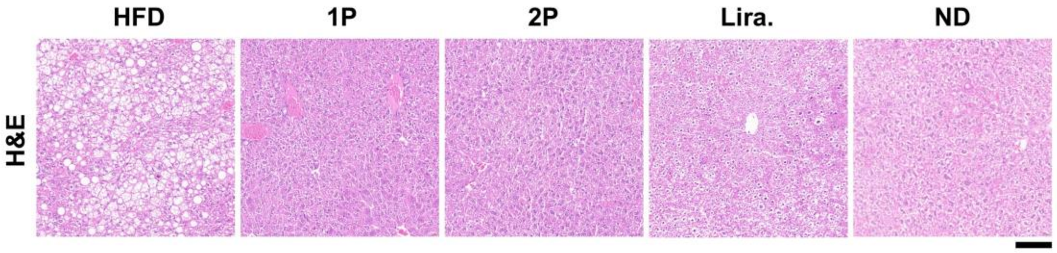

Figure S14. H&E staining of liver sections from HFD mice after 8 weeks treating with Peptides, scale bar 50 μm.

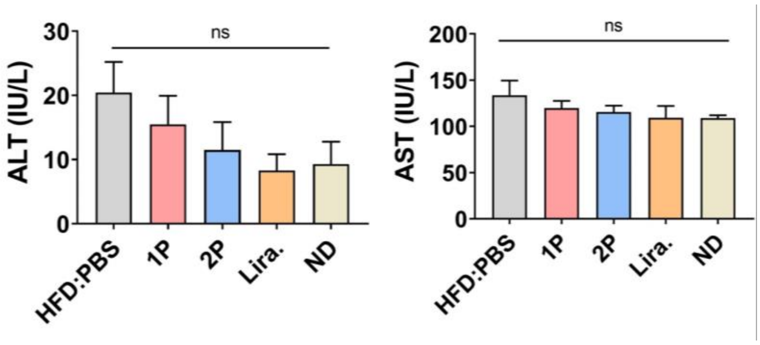

Figure S15. ALT and AST level in NAFLD-mouse serum after **1P** and **2P** treatment. Mean ± SD. *p < 0.05; **p < 0.01; ***p < 0.001; ns, no significance.

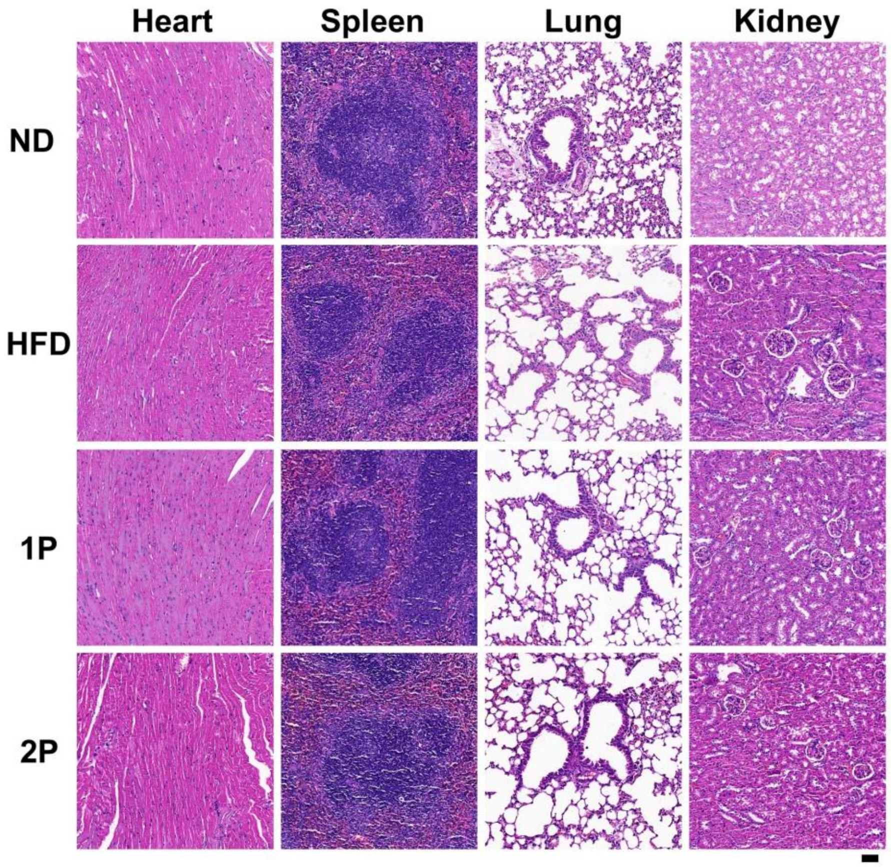

Figure S16. H&E staining of organ sections from HFD mice after 8 weeks treating with Peptides, scale bar 100 μm.

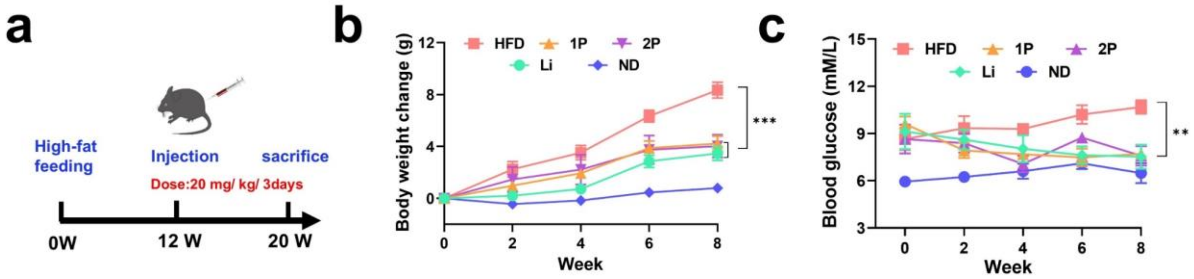

Figure S17. Evaluation of adiponectin self-assembling peptides in an obese mouse model. **(a)** schematic of NAFLD mouse model establishment and treatment protocol; **(b)** mouse body weight and **(c)** blood glucose levels during the treatment period. Mean ± SD. *p < 0.05; **p < 0.01; ***p < 0.001; ns, no significance.

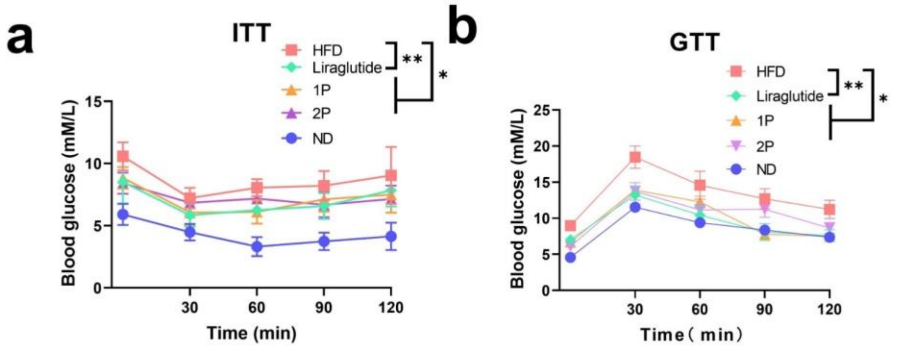

Figure S18. Assessment of adiponectin self-assembling peptide efficacy in an obese mouse model. **(a)** Insulin tolerance test in mice (n=5); **(b)** Glucose tolerance test in mice. Mean ± SD. *p < 0.05; **p < 0.01; ***p < 0.001; ns, no significance.

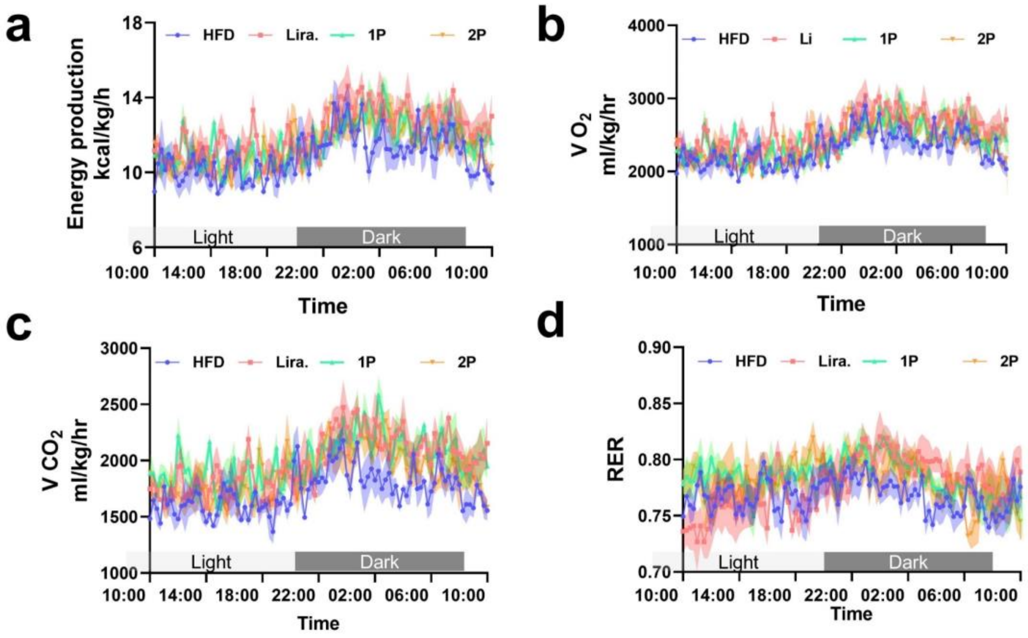

Figure S19. Metabolic cage study of peptide therapeutic efficacy. **(a)** energy expenditure of mice; **(b)** oxygen consumption of mice; **(c)** carbon dioxide production of mice; (d) respiratory entropy of mice (n=5).

6. Supplementary tables

Table S1. List of shRNA used in this study.

Name	Vector	Sequence (5`-3`)
shAdipor1-1	pLKO.1-mcherry-puro	CCGG-CGTCTATTGTCATTCAGAGAA-CTCGAG-TTCTCTGAATGACAATAGACG-TTTTTT
shAdipor1-2	pLKO.1-mcherry-puro	CCGG-GCCCACCATGCACTTTACTAT-CTCGAG-ATAGTAAAGTGCATGGTGGGC-TTTTTT
shAdipor1-3	pLKO.1-mcherry-puro	CCGG-CTGCTTGGTTTCGTGCTGTTT-CTCGAG-AAACAGCACGAAACCAAGCAG-TTTTTT
shAdipor1-4	pLKO.1-mcherry-puro	CCGG-CCCAAAGCTGAAGAAGAGCAA-CTCGAG-TTGCTCTTCTTCAGCTTTGGG-TTTTTT

Table S2. List of all qRT-PCR primers used in this study.

Gene name	Forward primer (5`-3`)	Reverse primer (5`-3`)
Soat2	GGGAGTGTTCCTGGTGTCTG	ATAGCATCAGCATGACCGGG
Hmgcs1	GGTGGCTATAAAGCTGCGGA	GGTGAAAGAGCCAAAGGGGAT
Pgc-1α	GTTGCCTGCATGAGTGTGTG	TAGAGACGGCTCTTCTGCCT
Hmgcr	GACTCTTGCGTCAACTTCAAGG	CAGGCTGTCTTTTGTCAACGA
Il-10	TCAAGGATGCACATCAAAAGGC	AGGCAGCAACTTCCTCCCT
Il-8	GACTCTTGCGTCAACTTCAAGG	CAGGCTGTCTTTTGTCAACGA
Tnfα	CCGGGAGAAGAGGGATAGCTT	TCGGACAGTCACTCACCAAGT
Inos	CTGGTGAAGGAACGGGTCAG	CCGATCATTGACGGCGAGAAT
Acox1	TTTTACCTTGCTTTCCTTGGCG	TGCCCAAGTGAAGGTCCAAA
Glut2	ACCGGGATGATTGGCATGTT	GAACACGTAAGGCCCAAGGA
Pparα	TATTCGGCTGAAGCTGGTGTAC	CTGGCATTTGTTCCGGTTCT
Hk2	CTGCTTTGGAGATCCGAGGG	AAGCAGGCGATCATATGCGA
Actin	GTAACCCGTTGAACCCCATT	CCATCCAATCGGTAGTAGCG
